# Two new species of *Yaginumaella*, Prószyński 1976 from Yunnan, China (Araneae, Salticidae)

**DOI:** 10.3897/zookeys.620.7895

**Published:** 2016-09-29

**Authors:** Wang Liu, Su-Fang Yang, Xian-Jin Peng

**Affiliations:** 1 College of Life Sciences, Hunan Normal University, Changsha, Hunan 410081, China

**Keywords:** Asia, description, diagnosis, jumping spider, taxonomy

## Abstract

The present paper deals with two new species of *Yaginumaella*, *Yaginumaella lushuiensis* **sp. n.** (female and male) and *Yaginumaella pseudoflexa* **sp. n.** (female and male). The male of *Yaginumaella lushuiensis* **sp. n.** differs from the related species *Yaginumaella flexa* Song & Chai, 1992 in ventral view of palpal organ. The female of *Yaginumaella lushuiensis* **sp. n.** differs from the related species *Yaginumaella urbanii* Żabka, 1981 by: 1) hoods locate at the anterior area of epigynum and far away from the copulatory openings; 2) epigynum about circular; 3) copulatory openings transverse. The male of *Yaginumaella pseudoflexa* **sp. n.** differs from the related species *Yaginumaella bulbosa* Peng, Tang & Li, 2008 in ventral view of palpal organ: 1) basal portion of embolus touches the margin of genital bulb. 2) distal portion of tibial apophysis covers the posterior margin of cymbium and far away from the margin of genital bulb. The female of *Yaginumaella pseudoflexa* **sp. n.** differs from the related species *Yaginumaella urbanii* Żabka, 1981 by: epigynum about as long as wide; hoods locate at the anterior area of the epigynum, above the outside area of the copulatory openings and far away from the copulatory openings. Photos of body and copulatory organs, line drawings of copulatory organs, as well as the locality map are provided. Descriptions of morphology are given.

## Introduction

*Yaginumaella* was established by Prószyński in 1979 with the type species *Yaginumaella ususudi*. A total of 42 species have been described mainly from subtropical Himalayan and Eastern Palaearctic areas (World Spider Catalog 2016). Żabka (1980, 1981) revised the diagnosis of the genus and described 27 new species. Up to now, 14 species have been recorded from China (Prószyn’ski 1979; Żabka 1980; Żabka 1981; Song and Chai 1992; Xie and Peng 1995; Yang et al. 1997; Peng et al. 2002; Zhu et al. 2005; Zhang and Zhu 2007).

While examining the specimens collected in the Gaoligong Mountains (Yunnan Province, Southwest China) by the Sino-American Expeditions (1998–2008), two new species of the genus *Yaginumaella* are found and described in this paper.

## Material and methods

All specimens were kept in 75% ethanol, examined, measured, and drawn with an Olympus SZX16 stereomicroscope and an Olympus BX53 compound microscope. Photos were taken with a digital camera Canon PowerShot G12 mounted on an Olympus SZX16. Compound focus images were generated using Helicon Focus software (3.10).

All measurements are given in millimeters. Leg measurements are given as: total length (femur, patella + tibia, metatarsus, tarsus). The abbreviations used in text include:

AER
 anterior eye row 


ALE
 anterior lateral eyes 


AME
 anterior median eyes 


CD
 copulatory ducts 


CO
 copulatory openings 


E
 embolus 


EFL
 length of eye 


H
 hood 


MOA
 median ocular area 


PER
 posterior eye row 


PLE
 posterior lateral eyes 


PME
 posterior median eyes 


S
 spermatheca 


TA
 tibial apophysis 


## Taxonomy

### Yaginumaella

Taxon classificationAnimaliaAraneaeSalticidae

Prószyński, 1976

Females in *Yaginumaella* have sclerotized blind hoods on epigyne, which are far away from the posterior edge, and differ in size and location. Copulatory ducts are of different length, with an internal ridge in the majority of species. The shape and size of spermathecae differ in various species.

Palpal organ in males rather simple, with end of embolus lying in a special groove on the ventral surface of cymbium usually more or less expanded laterally. Seminal receptacle thick. Cymbium densely covered with setae. Tibial apophysis robust and heavily sclerotized. Species differ in length and shape of embolus, bulb, and cymbium.

### Yaginumaella lushuiensis sp. n.

Taxon classificationAnimaliaAraneaeSalticidae

http://zoobank.org/379A5DDF-82DF-4CAA-9955-78D61F82690A

Figs 1–3 , 4–6 , 7–9 , 10–12

#### Type material.

***Holotype***: ♂, China: Yunnan: Lushui County: Pianma Township, 25.99363°N, 98.66651°E, 2470 m, 14 May 2005, C. Griswold. ***Paratypes***: 1♂,4♀, the same data as holotype.

#### Etymology.

The specific name refers to the type locality, Lushui County.

#### Diagnosis.

The male of the new species can be distinguished from all known congeneric species in ventral view of palpal organ by: embolus short, spatuliform; genital bulb without distinct posterior lobe; tibial apophysis extends to the top of genital bulb; embolus about 1/2 length of genital bulb. The female of the new species can be distinguished from all known congeneric species by: epigynum about circular; copulatory openings transverse.

#### Description.

**Male (holotype)**: Total length 4.60. Cephalothorax 2.15 long, 1.75 wide. Abdomen 2.35 long, 1.50 wide. Clypeus height 0.10. Carapace black-brown, with black margin, basal area of each eye, anterior and lateral margins of ocular area black. Thoracic region with two longitudinal dark bands. Marginal areas of carapace, anterior and lateral margins of ocular area densely covered with white hair; ocular area with thick dark brown hair; fovea short, longitudinal and black; cervical groove indistinct, radial groove dark brown. Sternum oval, covered with short brown hair, central area bulged, light yellow with gray edge. Clypeus narrow, height less than the radius of AME, light brown, promargin with white hair. Chelicerae dark brown, with brown hair, two promarginal and one retromarginal teeth (Fig. 6). Labium brown with brown hair, terminal area lightly colored. Palp and legs brown, legs with clear dark brown annuli. Eye sizes and interdistances: AER 1.50, PER 1.40, ALE 0.25, PLE 0.15, AME 0.50, EFL1.00. Measurements of legs: I 5.00 (1.50, 2.00, 1.00, 0.50), II 3.75 (1.00, 1.50, 0.75, 0.50), III 4.50 (1.50, 1.50, 1.00, 0.50), IV 4.25 (1.25, 1.50, 1.00, 0.50). Leg formula: 1342. Abdomen long oval, black to yellow brown, cardiac pattern long bar-shaped, muscular impressions clearly visible, posterior area of abdomen with six arc-shaped darker bands. Abdominal ventral: anterior area light brown, median area with one black longitudinal stripe, lateral areas with scattered grayish-black patches. Spinnerets brown.

Male palp (Figs 2–3, 4–5): tibia longer than wide in ventral view, with several long prolateral macrosetae in retrolateral view. Genital bulb with membrane structure. Embolus slender and about 1/2 length of genital bulb, originates from the position of 10:00 o’clock, its tip reaches to the position of 13:00 o’clock in ventral view. Bulb squat, median portion widest. Sperm ducts obvious, its diameter about 1/6 width of bulb.

**Figures 1–3.**
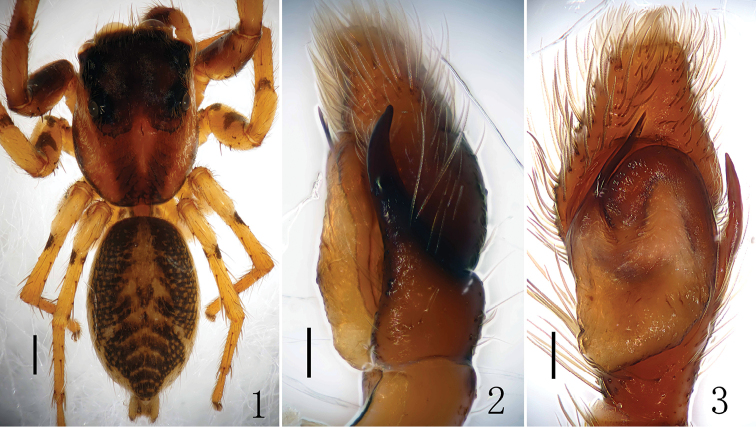
*Yaginumaella lushuiensis* sp. n. **1** male body, dorsal view **2** male palp, retrolateral view **3** male palp, ventral view. Scale bars: (**1**) 0.5 mm; (**2, 3**) 0.1 mm.

**Figures 4–6.**
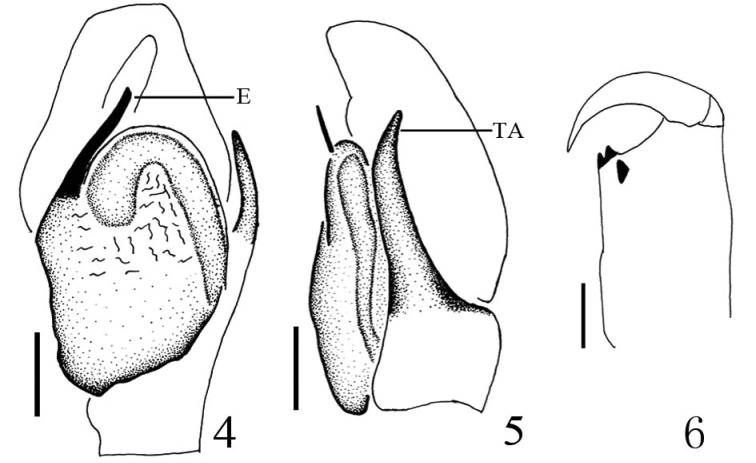
*Yaginumaella lushuiensis* sp. n. **4** male palp ventral view **5** male palp, retrolateral view **6** left chelicera, posterior view. Scale bars: 0.1 mm.

**Female**: Total length 5.00. Cephalothorax 2.40 long, 2.00 wide. Abdomen 2.60 long, 2.10 wide. Clypeus 0.15 high. Eye sizes and interdistances: AME 0.50, ALE 0.25, PLE 0.15, AER 1.60, PER 1.40, EL1.00. Legs yellow. Leg spinnation the same as male. Measurements of legs: I 4.85 (1.50, 1.85, 0.75, 0.75), II 3.85 (1.30, 1.30, 0.75, 0.50), III 4.75 (1.75, 1.3, 1.00, 0.75), IV 4.5 (1.25, 1.75, 1.00, 0.50). Leg formula: 1342. Other morphological characteristics the same as male except more pale in color.

Epigyne (Figs 8–9, 10–11) longer than wide, with two distinct anterior hoods. copulatory openings almost u-shaped, far away from the hoods. Copulatory ducts indistinct. Spermathecae big, squat, close to each other.

**Figures 7–9.**
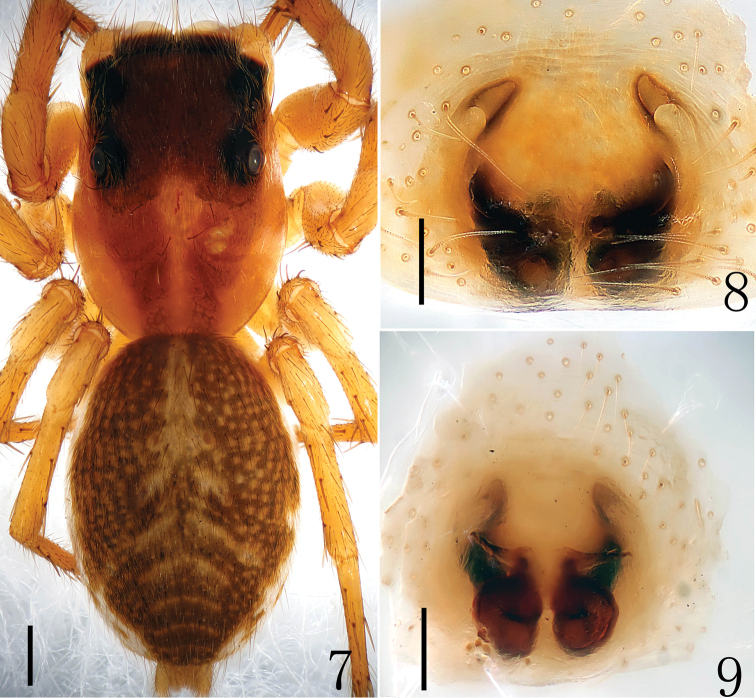
*Yaginumaella lushuiensis* sp. n. **7** female body, dorsal view **8** epigyne, ventral view **9** vulva, dorsal view. Scale bars: (**7**) 0.5 mm; (**8, 9**) 0.1 mm.

**Figures 10–12.**
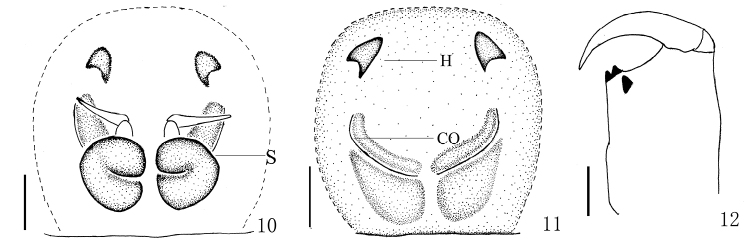
*Yaginumaella lushuiensis* sp. n. **10** vulva, dorsal view **11** epigynum, ventral view **12** left chelicera, posterior view. Scale bars: 0.1 mm.

#### Variation.

The male length 4.60–4.80 (n = 2) and the female length 4.80–5.60 (n = 4).

#### Distribution.

China (Yunnan).

### Yaginumaella pseudoflexa sp. n.

Taxon classificationAnimaliaAraneaeSalticidae

http://zoobank.org/0268AF85-0001-4F73-B2DA-D90232A13381

Figs 13–15 , 16–18 , 19–21 , 22–24

#### Type material.

***Holotype***: ♂, China: Yunnan: Lushui County: Pianma Township, 25.99363°N, 98.61704°E,1780 m, along the road in town 15 May 2005, G. Tang. ***Paratypes***: 1♂, 3♀, the same data as holotype.

#### Etymology.

The specific name is the combination of the Latin prefix “*pseudo*” and “*flexa*”, referring to the similarity of the new species to *Yaginumaella flexa* Song and Chai, 1992.

#### Diagnosis.

The male of this new species can be separated from all known congeneric species in ventral view of palpal organ by: basal portion of embolus touches the margin of genital bulb; distal portion of tibial apophysis covers the posterior margin of cymbium and far away from the margin of genital bulb. The female of this new species can be separated from all known congeneric species by: epigynum about as long as wide; copulatory openings almost parentheses-shaped; hoods locate above the outside area of the copulatory openings.

#### Description.

**Male (Holotype)**: Total length 5.40. Cephalothorax 2.60 long, 1.90 wide; Abdomen 2.80 long, 1.70 wide. Clypeus 0.15 high. Carapace brown, with black margin, basal area of each eye, anterior and lateral margins of ocular area black; Marginal areas of carapace and thoracic region with one longitudinal yellow brown band. Marginal areas of carapace, anterior margin of ocular area densely covered with white hair, sparsely covered with brown hairs; fovea short, longitudinal and reddish-brown; cervical groove indistinct, radial groove dark brown. Sternum scutiform, covered with short brown hair, dark brown with gray edge. Clypeus dark brown, with long brown setae. Promargin with dense hair. Chelicerae brown to dark brown, with brown hair; 2 promarginal and 1 retromarginal teeth (Figs 18). Labium dark brown, terminal brown, with dark brown hair. Endites base brown, terminal yellow brown, with dense dark brown hair. Legs yellow brown to dark brown; leg I dark brown, I and II spination v 2-2-2, I and II spination v 2-2. Measurements of legs: I 4.55 (1.65, 2.20, 1.00, 0.70), II 4.80 (1.60, 1.80, 0.80, 0.60), III 4.90 (1.60, 1.60, 1.00, 0.70), IV 5.40 (1.75, 1.75, 1.20, 0.70). Leg formula: 4321. Abdomen oval, yellow brown, with 6 muscular impressions; lateral areas with two grayish-black longitudinal stripes and scattered black diagonal patches. Posterior area of abdomen with arc-shaped and dentiform dark bands; ventral yellowish-white, with scattered grayish-black patches; median area with one black longitudinal stripe, lateral areas with scattered black diagonal patches. Spinnerets black-brown.

Male palp (Figs 14–15, 16–17): tibia longer than wide in ventral view, with several long prolateral macrosetae in retrolateral view. Genital bulb with membrane structure. Embolus slender and sinuous, nearly as long as genital bulb, originates from the position of 9:00 o’clock, its tip reaches to the position of 14:00 o’clock in ventral view. Bulb squat, median portion widest. Sperm ducts obvious, its diameter about 1/3 width of bulb.

**Figures 13–15.**
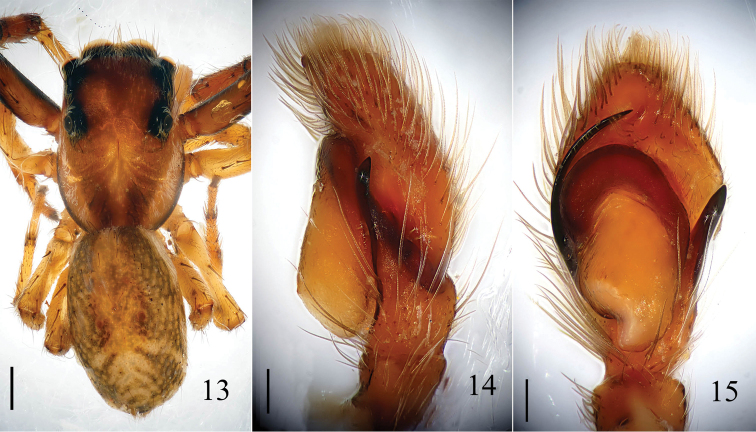
*Yaginumaella pseudoflexa* sp. n. **13** male body, dorsal view **14** male palp, retrolateral view **15** male palp, ventral view. Scale bars: (**13**) 0.5 mm; (**14, 15**) 0.1 mm.

**Figures 16–18.**
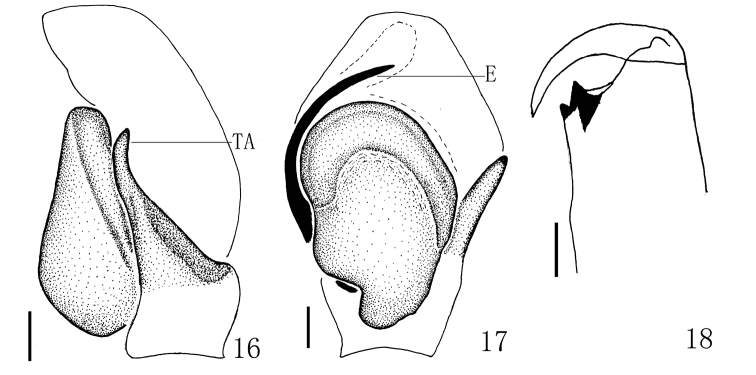
*Yaginumaella pseudoflexa* sp. n. **16** male palp, retrolateral view **17** male palp, ventral view **18** left chelicera, posterior view. Scale bars: 0.1 mm.

**Female**: Total length 5.40, Cephalothorax 2.40 long, 1.85 wide. Abdomen 3.00 long, 1.90 wide. Clypeus 0.15 high. Eye sizes and interdistances: AME 0.50, ALE 0.30, PLE 0.25, AER 1.65, PER 1.55, EFL1.00. Measurements of legs: I 4.30 (1.40, 1.70, 0.70, 0.50), II 3.90 (1.30, 1.05, 0.06, 0.50), III 4.80 (1.40, 1.70, 0.90, 0.80), IV 5.20 (1.60, 1.90, 1.00, 0.70). Leg formula: 4312. Other morphological characteristics the same as male, but lightly colored.

Epigyne (Figs 20–21, 22–23) as long as wide, with two distinct anterior hoods. Copulatory openings almost parentheses-shaped, far away from the hoods. Copulatory ducts thick and sinuous. Spermathecae big, squat, close to each other.

**Figures 19–21.**
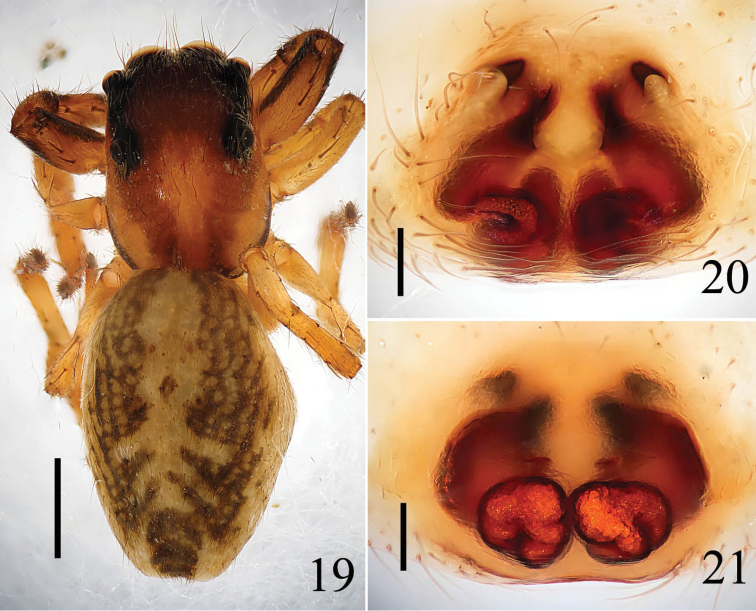
*Yaginumaella pseudoflexa* sp. n. **19** female body, dorsal view **20** epigynum, ventral view **21** vulva, dorsal view. Scale bars: 0.1 mm.

**Figures 22–24.**
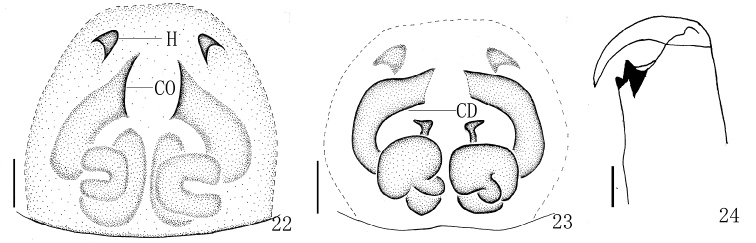
*Yaginumaella pseudoflexa* sp. n. **22** epigynum, ventral view **23** vulva, dorsal view **24** left chelicerae, posterior view. Scale bars: 0.1 mm.

**Figures 25.**
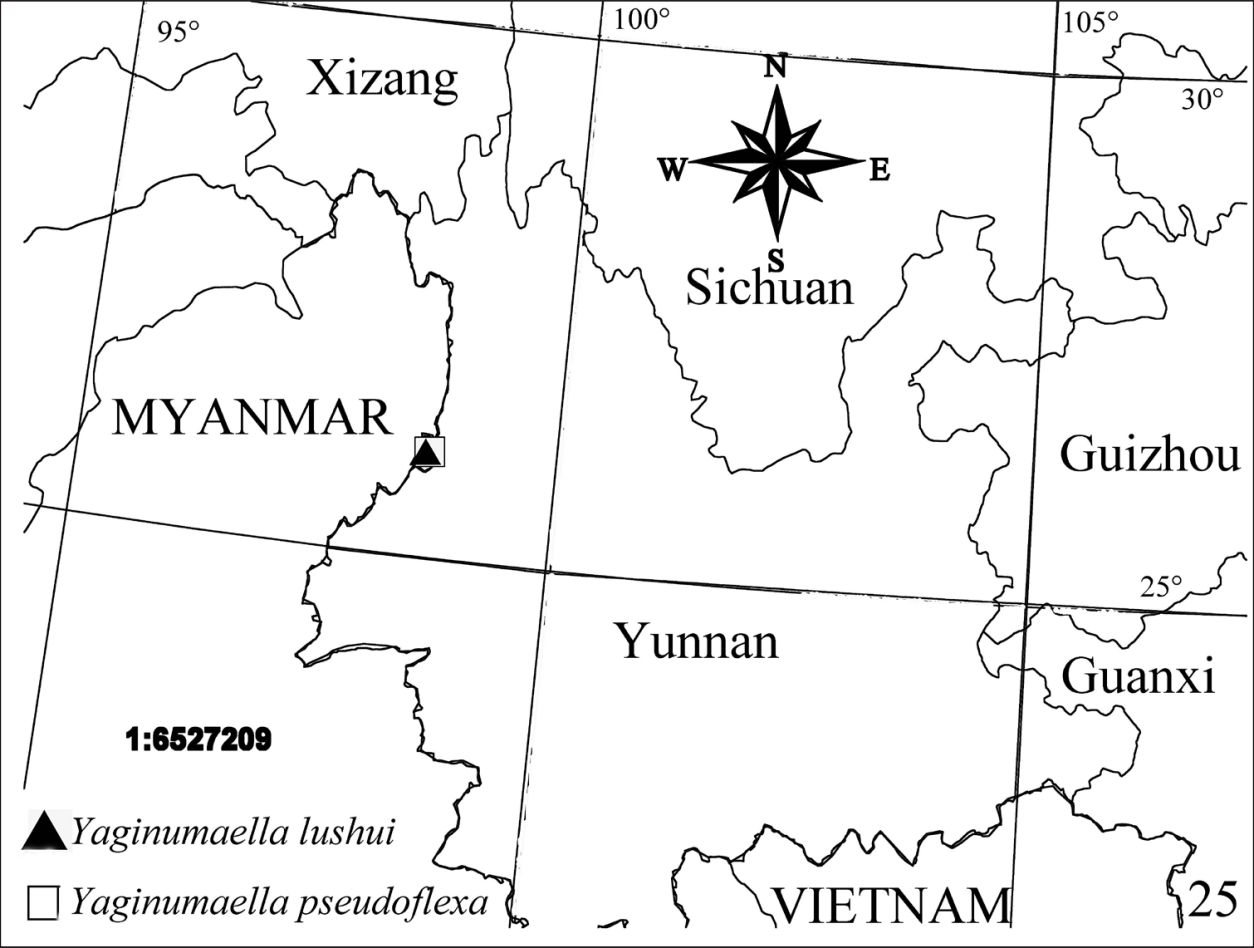
Localities of new *Yaginumaella* species from China.

#### Variation.

The male length 4.30–5.40 (n = 2) and the female length 4.80–5.80. (n = 3).

#### Distribution.

China (Yunnan).

## Supplementary Material

XML Treatment for Yaginumaella

XML Treatment for Yaginumaella lushuiensis

XML Treatment for Yaginumaella pseudoflexa
